# Paper 1: a systematic synthesis of narrative therapy treatment components for the treatment of eating disorders

**DOI:** 10.1186/s40337-022-00635-5

**Published:** 2022-09-08

**Authors:** Lauren Heywood, Janet Conti, Phillipa Hay

**Affiliations:** 1 School of Psychology, Western Sydney University, Sydney, Australia; 2 Translational Health Research Institute, Western Sydney University, Sydney, Australia; 3 School of Medicine, Western Sydney University, Sydney, Australia

**Keywords:** Eating disorders, Narrative therapy, Treatment components, Systematic review

## Abstract

**Background:**

There are presently a number of eating disorder treatment interventions that have a research-evidence base to support their effectiveness. However, rates of attrition and treatment outcomes demonstrate that there is no one-size fits all for the treatment of eating disorders. Narrative therapy is a promising, but under-researched, intervention for the treatment of eating disorders (EDs). The aim of this study was to conduct a narrative synthesis of the literature to explore the content and use of narrative therapy in the treatment of EDs.

**Method:**

Data were extracted from 33 eligible included studies following systematic search of five data bases. Data included aims and objectives, sample characteristics, treatment details and components of narrative therapy, which informed the narrative synthesis. The study is reported according to the Preferred Reporting items for Systematic Reviews and Meta-Analyses (PRISMA) guidelines.

**Results:**

Narrative therapy interventions for EDs consisted of several components including the narrative worldview; unpacking the problem story; finding, thickening the meaning and performance of stories hidden by the problem story; and safety considerations. A notable proportion of the extracted articles discussed components of unpacking problem stories, and finding and re-authoring openings (or unique outcomes) that were hidden by problematic stories. Relatively fewer papers discussed the processes by identity shifts were performed or lived out, including in the eating practices of those with a lived ED experience. Furthermore, few papers addressed how therapists established client safety within the narrative framework when working with EDs.

**Conclusion:**

This narrative synthesis found that narrative practitioners utilise a variety of narrative therapy techniques in working with individuals with a lived ED experience. The current literature has emphasis on specific narrative therapy techniques used in ED treatments, with some aspects of the narrative worldview and safety considerations left undiscussed. Additional research is needed to explore how identity shifts in narrative therapy are performed and lead to measurable behavioural changes, and to consider how safety considerations can be established within the narrative worldview.

**Plain English summary:**

There are a number of psychological therapies for eating disorders that have research evidence-base. These treatments, however, do not work for everyone as indicated by drop-out rates and eating disorders running a severe and enduring course for some people. Narrative therapy is a therapeutic intervention that has been reported as a promising intervention for people with an eating disorder. The focus of narrative therapy is to engage the person in finding identities hidden by problem-saturated identities and in the performance of these hidden identities. In this review, we have explored the use of narrative therapy for eating disorders to identify what aspects of this therapy are currently being used and which components are less referenced in the literature. Findings from this study support the need for further research into narrative therapy components in the treatment of eating disorders, particularly how hidden identities are performed and safety considerations are integrated into this therapeutic intervention.

**Supplementary Information:**

The online version contains supplementary material available at 10.1186/s40337-022-00635-5.

## Background

Eating Disorders (EDs) are defined in the Diagnostic Statistical Manual of Mental Disorders–Fifth Edition (DSM-5) [1] as a cluster of behaviours designed to control weight, which have negative impacts on physical and psychological functioning. The DSM-5 identifies several diagnostic categories of EDs including, but not limited to, Anorexia Nervosa (AN), Bulimia Nervosa (BN) and Binge Eating Disorder (BED).

The restrictive and purging behaviours associated with AN and binge purging behaviours in BN can result in substantial and life-threatening medical conditions that can have long-term physiological consequences [1, 2]. Individuals with AN have a death rate that is six times higher than that of the general population and the crude mortality rate (CMR) is estimated to be at 5% and 2% per decade for AN and BN respectively [1, 3]. This increased mortality rate is thought to be partially due to the elevated suicide rates in individuals with eating disorders, including 7% increased risk for individuals with BN and 12% for those with AN [1].

AN and BN also have high comorbidity with other psychiatric disorders, including anxiety and obsessive–compulsive disorders [4, 5], depression [6], and cluster B personality disorders (anxious-avoidant, obsessive–compulsive, and dependant) [7, 8]. Additionally, individuals who have experienced significant weight loss or malnutrition may experience symptoms including low mood, difficulties sleeping, social withdrawal, decreased libido and increased irritability as a result of the physical effects associated with starvation [9, 10]. BED is thought to be associated with significant psychiatric comorbidity which is linked to the severity of binge-eating, with the most common disorders being mood disorders (i.e., bipolar disorder and depression), anxiety disorders and substance use disorders [1].

### Treatments and outcomes

Treatments for EDs have varied significantly across time and only a limited range of interventions have been empirically evaluated or delivered in a specialised format [11–15]. Current treatments for EDs are facilitated in inpatient, day and outpatient settings. There is evidence that CBT-based programs have found to be effective in the treatment of BN, with manualised CBT programs showing significant improvements in weight gain and decreased eating disorder (ED) symptomatology [13]. For adult AN, the Maudsley Model of Treatment for Adults [16], psychodynamic approaches [17], and Specialist Supportive Clinical Management (SSCM) [14] have been found to be effective in symptom reduction for adult AN. For adolescent AN, Maudsley Family Therapy and Family-Based Therapy (FBT) [18] have been found to be effective in symptom reduction. Despite this, there is no one treatment modality that is regarded as best practice for the treatment of AN [14]. Furthermore, psychological treatments for EDs have not been shown to be consistently more effective than treatment as usual, and no specific treatment is consistently more successful than others [15, 19, 20].

Further research has shown that treatments that focus primarily on eating behaviour and weight restoration may inadequately address the broader needs and preferences of those with a lived ED experience [21–25]. These challenges in determining best practice for treatment of EDs may be partially explained by the complexities characteristic of EDs. Many individuals with EDs report pervasive negative self-concepts, which can lead them to feel hopeless about their capacity to recover from an ED and undermine treatment engagement [26]. EDs may also be perceived by individuals as protective due to providing a sense of control, structure and/or achievement [27]. Individuals may begin to rely on and/or aspire to ongoing weight loss as the primary way of defining their identity and/or to reinstate a sense of self that may be due, in part, to others praising them when they first begin to lose weight [28–30]. As such, many individuals may struggle to conceptualise their lived experience of an ED as problematic, which is known as the egosyntonic features of the illness [23, 31].

Research has also indicated that many individuals feel ambivalent about engaging in ED treatments [32–34]. This may be due, in part, to fear and uncertainty related to the potential loss of the ED [35]. Treatments have been described as traumatic for some [36], and the loss of the ED identity as traumatic for others [37]. Some individuals have conceptualised EDs as an enemy and a friend, and as somehow both separate and intrinsic to their own identity [38–40]. Likewise, other individuals have spoken about their experience as constructing and defending different ‘AN selves’, where recovery from AN involved a loss of identity or selves driven by perfection, sensitivity, competitiveness and protectiveness [41].

The perspectives of individuals on what constitutes ‘recovery’ from an ED ranges from ‘completely recovered’ to those who feel that the illness will be something they struggle with for the remainder of their lives [23, 42]. Furthermore, individuals may experience recovery as going beyond symptom improvement, such as improving their overall quality of life [43] and gaining a sense of identity outside the ED [37, 39].

The complexities associated with recovery from EDs highlight the need to consider how therapeutic interventions assist individuals in navigating issues of identity [25]. A need has been identified for treatments to more comprehensively focus on dismantling the ED-dominant identity and exploring the individual’s sense of self outside of the ED [34, 43]. Individuals receiving treatment for BN have also emphasised the importance of person-centred practise that prioritises the creation of a ‘meaningful life’ [33, 44–46]. Likewise, there is a growing body of literature to support the use of BED interventions that focus on developing self-compassion, rather than focusing exclusively on weight change [46–49].

As such, development and further research of new and emerging treatments for EDs is required in the hope of improving recovery rates, particularly in outpatient settings [9, 50]. There is an expressed need by those with lived ED experience for interventions to explore questions of identity, including who am I outside of the ED identity? [31, 37]. This is particularly relevant when considering the perspective that people live out or perform the meaning of their identity narratives or the stories they tell about themselves [51, 52].

### Narrative therapy

Narrative therapy, as developed by Michael White and David Epston, has been proposed as a therapeutic intervention for a range of psychological difficulties, including EDs [53–55]. The key treatment components of narrative therapy are first, deconstruction and externalisation of the problem story, and second finding, thickening, and performing the meaning of identity stories hidden by dominant, problem-saturated stories [53, 54, 56–64]. Narrative therapy is positioned within the broad philosophical movements of social constructionism and poststructuralism [53], which understand that the meanings a person makes of their life are shaped by social and cultural contexts within which they live. These contexts give rise to certain discourses, or taken-for-granted “truths” that are then taken up by individuals to construct versions of reality that are organised in narrative form, or as stories [60, 65]. Narrative therapy proposes that a person’s identity is constructed in storied form and these identity stories guide how they think, feel and act in their lives [66].

Deconstruction or unpacking the meaning of dominant identity stories in narrative therapy is theoretically underpinned by post-structuralist philosophies, particularly the work of Michel Foucault [67]. The therapeutic practice of deconstruction of problematic stories is proposed to be instrumental in releasing self knowledges that were previously hidden by dominant storylines. These released self-knowledges are then available to the person to piece together an identity built on a valued sense of themselves [68]. Within a narrative worldview, a person’s identity is understood as multi-storied where their relationship with the problem is one of the many stories of their life. When a problem narrative dominates (for example the ED narrative), other stories of self are obscured. We have termed these stories, ‘hidden stories’ (as named by Daphne Hewson; personal communication) as they are hidden from view when the ED identity story dominates. These narratives, and the language that is constitutive of them, do not merely describe a person to themselves and others. Narrative therapy posits that individuals ‘perform the meaning’ of these stories [53]. That is, the stories we believe in and adopt as explanations of our reality, shape how we interact in everyday life.

There are three primary processes of narrative therapy. First, the person, family or community are invited to map the effects of the problem on their life, relationships and their identity [54]. This includes deconstruction or unpacking of meaning of problematic storylines and externalisation, where the problem is linguistically separated from the person’s identity [53, 56–59] and named on the person’s experience-near terms.

The second process of narrative therapy is known as finding unique outcomes [60] and focuses on working with the person to find and reveal identity narratives that have been hidden by the dominant problem [54]. Finding unique outcomes is facilitated by relative influencing questioning to map the influence of the person over the problem that traces ways they have responded to and stood up to the problem [61, 62] and ‘double listening’ [63] to identify what is absent but implicit in the problem story that speaks to the person’s preferred and valued stories [64]. In doing so, therapy focuses on ‘re-authoring conversations’ [54] that generate identity narratives that align more comprehensively with a person’s life, who they understand themselves to be, and their valued sense of themselves.

The final primary process of narrative therapy is thickening the meaning of hidden storylines that have been buried under the problem story through reviewing the history of the person’s influence over the problem and ways that their actions are inconsistent with the problem story. This leads to new meanings being made for old stories or ‘new-old stories’ [64]. Unique outcomes are further thickened by exploring the link to a person’s values, hopes, dreams, intentions, and possible future [62, 69]. The presence of significant others is invited through the practice of ‘re-membering conversations’ that aim to provide rich identity accounts of memories of events where the person displayed particular values, skills or traits consistent with the revealed storylines [64] Narrative therapy also utilises therapeutic letter writing for the purposes of extending the therapeutic conversation between sessions, with a particular focus on documenting and thickening unique outcomes through prompting further reflections [53, 60]. Letters may also be sent to family members or loved ones, to invite them to attend sessions or celebratory rituals, termed ‘reflecting teams’ [53].

Therefore, narrative therapy has scope to comprehensively address questions of identity [53, 59], which have been identified to be important to the experiencing person across qualitative studies in ED treatments [25]. Despite this, there are few comprehensive systematic reviews on the content or treatment outcomes studies of narrative therapy conducted to treat EDs. Likewise, there does not currently exist a manualised approach to using narrative therapy in the treatment of EDs. Manualising narrative therapy is seemingly counter-intuitive to the narrative approach that is designed to be person-centred with the worldview that the person is the expert of their life and therefore need not be treated by being fitted into a pre-existing treatment framework or model. Nevertheless, there is a need to have a greater understanding of the components proposed by those who have expertise in the treatment of EDs with narrative therapy to inform future research into narrative therapy treatments for EDs.

### The current study

The aim of this narrative synthesis was to synthesise information concerning the content of narrative interventions for individuals who experience EDs. It sought to understand key aspects, content, and techniques of narrative therapy interventions in EDs in order to inform their future development. Specific techniques examined in the narrative synthesis included components of the narrative worldview, deconstruction of and mapping the effects of the problem, discovering unique outcomes and hidden narratives, and thickening the meaning of new storylines.

## Method

This narrative synthesis was registered with Open Science (10.17605/OSF.IO/2KU3C) and was conducted in line with the Preferred Reporting Items for Systematic Reviews and Meta-Analyses (PRISMA) guidelines [70].

### Identification and selection of studies

The electronic databases searched were PsychINFO, MEDLINE, EMBASE, SocIndex and ProQuest Dissertations and Theses (grey literature). References were also identified and nominated by author JC’s personal library of narrative therapy resources. The dates searched included all dates from 1979 to the 4^th^ of July 2021. A search strategy was developed in consultation with a librarian and key search terms were (anorexi* OR anore*) OR (bulimi* or bulim*) OR (eat* or eating) OR (binge eat*) AND (intervention* OR treatment* OR therapy OR counsel*) AND (narrative).

Articles were included if they met the following criteria: (a) Published in English, (b) Focused on the content of narrative therapy interventions (including specific details of said content); (c) Included a sample of individuals in treatment for any ED, including books that describe narrative therapy interventions and case studies that use illustrative examples. Studies were excluded if they were: (a) Review papers, (b) Not published in English, (c) If full text was unavailable, or (d) Did not describe narrative therapy interventions as a treatment for any ED.

### Study selection

One reviewer (LH) ran the identified search terms across all electronic databases, including grey literature. Another reviewer (JC) identified relevant articles from their personal library of narrative therapy resources. All texts were then combined and duplicates removed. The title and abstract of each paper were individually evaluated by two reviewers (LH and JC) for their adherence to inclusion criteria and any discrepancies were resolved by a third reviewer (PH). The full text of publications were obtained if they met criteria and any unavailable full texts were excluded. The first reviewer (LH) assessed eligibility of full-text references for inclusion, with assistance from the second reviewer (JC) regarding any uncertainties.

### Quality assessment

All included publications were assessed independently by two reviewers (LH and JC) using independent quality appraisal assessment tools adapted from the Downs & Black Checklist (1998) [71] and the Joanna Briggs Institute’s Checklist for Text and Opinion (2015) [72]. Articles were rated based on a 10-item assessment criteria which included; reference to extant literature, clarity of hypothesis/aim/objective, description of main outcomes, reports of characteristics of participants, appropriate recruitment, description of intervention components, identification of main findings, logical presentation of conclusions, explanation for discrepancies with pre-existing literature, and evidence of ethical practice. The remaining one book text was rated based on six item assessment criteria which included: definition and quality of the source of opinion, interests of the population posited as central focus, logical presentation of conclusions, reference to extant literature, and provision of explanation for discrepancies with pre-existing literature. Each criterion was scored on a 3-point scale, where 0 = no, 1 = unclear/partial, and 2 = yes. Any discrepancies in ratings given by reviewers were resolved through discussion. All the studies meeting selection criteria were retained as it was thought that each study provided relevant qualitative data, regardless of their quality assessment score. See Additional file 1 (Tables S1 and S2) for the quality appraisal ratings for each of the included references.

### Results

**Fig. 1:**
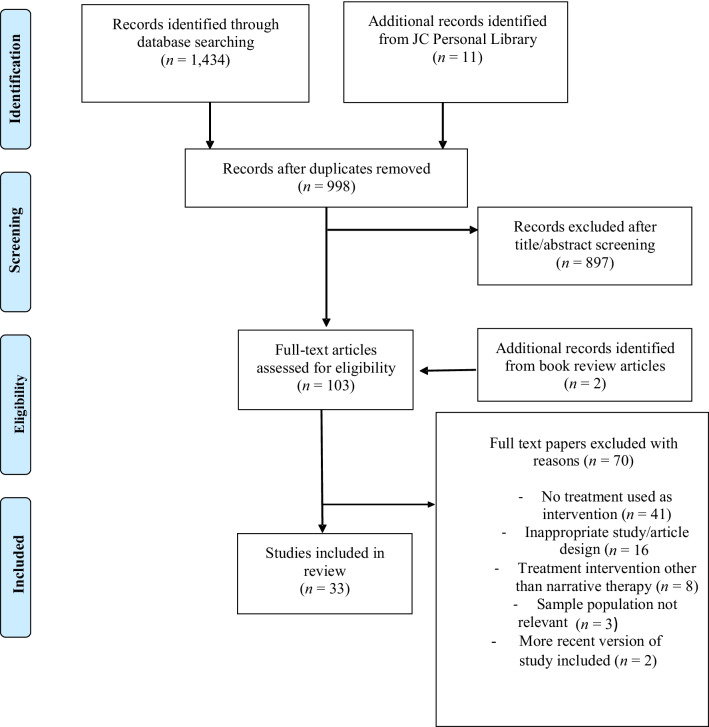
Flow chart of search strategy

A total of 1434 articles were identified from the online database search, with an additional 11 from JC’s personal library. Duplicates were removed and 998 references remained. Following title and abstract screening according to exclusion and inclusion criteria, and the addition of two records identified from book review articles, 103 articles remained. Full-text screening was then conducted, and the remaining 33 texts were included (see Fig. 1).

### Study characteristics

The 33 studies were summarised and data was extracted with regard to the following: aims and objectives, sample characteristics, and treatment details (see Additional file 1: Table S3). Of the 33 included papers, 14 consisted of case studies with exemplar therapy transcripts (*n* = 1). Ten of the articles included case studies of between two to four clients, and six of the included papers reported on five or more client cases. Three papers did not report the number of clients from which their data was obtained. The origins of the samples varied, with the most being based in Australia (*n* = 8), followed by six from Canada, four from New Zealand, four from the United States, and three from England. Likewise, Hong Kong, Israel, Greece, and Norway each contributed one article. Thirty-one of the articles were originally published in English, with one written in Norwegian [73] and the other in Hebrew [74] prior to translation.

Across all included studies, the age range of clients spanned from eight to fifty years old. The majority of clients were female and were seen due to eating concerns. Clients were seen in various treatment contexts, including community centres, public hospitals, outpatient day programs, and most commonly, in outpatient settings (*n* = 18). Similarly, treatment sessions were provided in both individual and family settings (*n* = 28) and group formats (*n* = 5).

### Quality appraisal findings

The quality appraisal revealed that a large proportion of the studies provided substantive reference to the extant literature (*n* = 26), described the hypothesis/aim/objective of the study (*n* = 25), defined the characteristics of their sample (*n* = 21), adopted a logical conclusion/position (*n* = 25), defended any incongruence with the pre-existing literature (*n* = 17) and discussed the components of the intervention described (*n* = 31). A lower number of papers were assessed as being representative of the sample size, with five articles obtaining a score of two and 25 studies scoring a one on the quality assessment rating. The most frequent reason for a low score on the quality appraisal criteria was a lack of direct evidence for ethics approval, with only two of the papers citing a process of applying for ethics approval. An additional 30 articles appeared committed to practising in an ethical manner, however, did not explicitly mention any approval by an ethics committee.

The book text was assessed according to modified quality appraisal criteria. Results indicated that this text clearly defined the source of opinion which had standing in the field of expertise, spoke to the interests of the relevant population, presented logical conclusions/positions, and referred to the extant literature. Likewise, the book was assessed as partially defending any logical incongruence with pre-existing literature.

### Synthesis of results

All articles were further analysed to explore which components of narrative therapy were included in each intervention. The treatment components for each study can be seen in Table 1 and have been analysed according to the following categories:

(A) Narrative worldview,
(B) Unpacking of problem stories,
(C) Finding hidden and new stories,
(D) Thickening revealed and new stories, and
(E) Safety considerations

**Table 1:** Components of narrative therapy intervention for eating problems/disorders

Author/s (Year)	A. narrative worldview	B. Unpacking of problem stories			C. Finding hidden and new stories, and D. Thickening revealed and new stories				E. Safety considerations
		Deconstruction	Externalisation	‘Experience-near’ naming	Finding openings to hidden narratives	Thickening hidden stories	Creative and letter writing	Outsider witnessing	
Beaudoin (2020) [72]		$\checkmark$		$\checkmark$	$\checkmark$	$\checkmark$			
Borden (2007) [73]	$\checkmark$	$\checkmark$	$\checkmark$	$\checkmark$	$\checkmark$	$\checkmark$		$\checkmark$	
Brown (2018) [74]	$\checkmark$	$\checkmark$	$\checkmark$		$\checkmark$				
Brown, Weber & Ali (2008) [75]	$\checkmark$	$\checkmark$	$\checkmark$		$\checkmark$	$\checkmark$			
Courtney & Williams (2000) [76]	$\checkmark$	$\checkmark$	$\checkmark$	$\checkmark$	$\checkmark$	$\checkmark$	$\checkmark$	$\checkmark$	
Craggs & Reed (2007) [77]	$\checkmark$	$\checkmark$							
Davidson & Birmingham (2001) [78]	$\checkmark$		$\checkmark$				$\checkmark$		
Dallos (2004) [79]	$\checkmark$	$\checkmark$	$\checkmark$			$\checkmark$			
Dennstedt (2010) [80]	$\checkmark$	$\checkmark$							
Epston, Morris & Maisel (1995) [81]		$\checkmark$	$\checkmark$	$\checkmark$	$\checkmark$	$\checkmark$	$\checkmark$	$\checkmark$	
Golan (2013) [71]	$\checkmark$*	$\checkmark$	$\checkmark$		$\checkmark$	$\checkmark$	$\checkmark$	$\checkmark$	$\checkmark$*
Howells (2009) [82]		$\checkmark$*	$\checkmark$	$\checkmark$	$\checkmark$	$\checkmark$	$\checkmark$	$\checkmark$	
Ibrahim & Tchanturia (2018) [83]	$\checkmark$	$\checkmark$	$\checkmark$		$\checkmark$		$\checkmark$	$\checkmark$	
Ingamells (2016) [84]	$\checkmark$		$\checkmark$	$\checkmark$	$\checkmark$	$\checkmark$			
Kantor & Levine (2000) [85]	$\checkmark$	$\checkmark$	$\checkmark$	$\checkmark$	$\checkmark$	$\checkmark$		$\checkmark$	$\checkmark$
Kraner & Ingram (1997) [86]	$\checkmark$	$\checkmark$	$\checkmark$	$\checkmark$	$\checkmark$	$\checkmark$		$\checkmark$	
Lainson (2016) [87]	$\checkmark$	$\checkmark$	$\checkmark$	$\checkmark$	$\checkmark$	$\checkmark$		$\checkmark$	
Lainson (2019) [88]	$\checkmark$	$\checkmark$	$\checkmark$	$\checkmark$	$\checkmark$	$\checkmark$			
Lock, Epston & Maisel (2004) [89]	$\checkmark$	$\checkmark$	$\checkmark$		$\checkmark$		$\checkmark$		
Lock, Epston, Maisel & de Faria (2005) [90]	$\checkmark$	$\checkmark$	$\checkmark$	$\checkmark$	$\checkmark$				
Lundby (2014) [70]	$\checkmark$	$\checkmark$	$\checkmark$	$\checkmark$	$\checkmark$	$\checkmark$	$\checkmark$		
Maisel, Epston & Borden (2004) [52]	$\checkmark$	$\checkmark$	$\checkmark$	$\checkmark$	$\checkmark$	$\checkmark$	$\checkmark$	$\checkmark$	$\checkmark$
Nylund (2002) [91]	$\checkmark$	$\checkmark$	$\checkmark$		$\checkmark$	$\checkmark$	$\checkmark$	$\checkmark$	
Pedersen (2016) [92]	$\checkmark$	$\checkmark$	$\checkmark$	$\checkmark$	$\checkmark$	$\checkmark$	$\checkmark$	$\checkmark$	
Robbins & Pehrsson (2009) [93]	$\checkmark$	$\checkmark$	$\checkmark$	$\checkmark$	$\checkmark$	$\checkmark$	$\checkmark$	$\checkmark$	
Russell (2007) [94]	$\checkmark$	$\checkmark$	$\checkmark$	$\checkmark$	$\checkmark$	$\checkmark$			
Scott, Hanstock & Patterson-Kane (2013) [95]		$\checkmark$	$\checkmark$	$\checkmark$	$\checkmark$	$\checkmark$			$\checkmark$
Tsun on-Kee (2011) [96]		$\checkmark$	$\checkmark$		$\checkmark$	$\checkmark$			
Weber (2007) [97]	$\checkmark$	$\checkmark$	$\checkmark$	$\checkmark$	$\checkmark$				$\checkmark$
Weber, Davis & McPhie (2006) [98]	$\checkmark$	$\checkmark$	$\checkmark$	$\checkmark$				$\checkmark$	$\checkmark$
White (1986) [61]	$\checkmark$	$\checkmark$	$\checkmark$		$\checkmark$				
Zimmerman & Dickerson (1994) [99]	$\checkmark$	$\checkmark$	$\checkmark$	$\checkmark$	$\checkmark$	$\checkmark$			
White (1991) [62]	$\checkmark$	$\checkmark$	$\checkmark$	$\checkmark$	$\checkmark$	$\checkmark$		$\checkmark$	

*Brief reference to this component

#### Narrative worldview

Aspects of the narrative worldview were expressed in 28 of the identified articles. Pedersen [75] defined narrative therapy as a belief system or philosophy, rather than being limited to a collection of psychological techniques. This idea was mirrored across many of the articles identified in the search, with 18 references describing how narrative therapy encapsulates core tenants of social constructionism. This positioned narrative therapy as intrinsically political [53, 59, 61, 73, 75–88]. In particular, the narrative worldview was depicted by the studies as including the stance of the therapist, the client positioned as the expert on their life, and ‘the problem is the problem’ [59, 85]. Likewise, the narrative worldview conceptualised identity as multi-storied, used non-pathologising language, and considered the person’s readiness for change. The specific results of which articles contained reference to these components of the narrative worldview can be found in Table 2.

**Table 2:** Components of narrative therapy worldview

Author/s (Year)	Social constructionism	Therapist stance	The person is the expert	the problem is the problem	Identity is multi-storied	Perform the meaning	Language	Readiness for change
Borden (2007) [73]	$\checkmark$	$\checkmark$	$\checkmark$		$\checkmark$	$\checkmark$	$\checkmark$	
Brown (2018) [74]	$\checkmark$	$\checkmark$	$\checkmark$	$\checkmark$			$\checkmark$	
Brown, Weber & Ali (2008) [75]	$\checkmark$			$\checkmark$	$\checkmark$	$\checkmark$	$\checkmark$	
Courtney & Williams (2000) [76]	$\checkmark$		$\checkmark$				$\checkmark$	
Craggs & Reed (2007) [77]		$\checkmark$			$\checkmark$		$\checkmark$	
Dallos (2004) [79]		$\checkmark$		$\checkmark$			$\checkmark$	
Davidson & Birmingham (2001) [78]							$\checkmark$	$\checkmark$
Dennstedt (2010) [80]		$\checkmark$	$\checkmark$				$\checkmark$	
Golan (2013) [71]		$\checkmark$	$\checkmark$			$\checkmark$*	$\checkmark$	$\checkmark$
Ibrahim & Tchanturia (2018) [83]		$\checkmark$	$\checkmark$		$\checkmark$		$\checkmark$	$\checkmark$
Ingamells (2016) [84]		$\checkmark$	$\checkmark$	$\checkmark$	$\checkmark$	$\checkmark$	$\checkmark$	
Kantor & Levine (2000) [85]	$\checkmark$						$\checkmark$	
Kraner & Ingram (1997) [86]	$\checkmark$		$\checkmark$		$\checkmark$		$\checkmark$	
Lainson (2016) [87]	$\checkmark$	$\checkmark$	$\checkmark$			$\checkmark$	$\checkmark$	
Lainson (2019) [88]	$\checkmark$		$\checkmark$	$\checkmark$	$\checkmark$	$\checkmark$	$\checkmark$	
Lock, Epston, Maisel & de Faria (2005) [90]	$\checkmark$		$\checkmark$	$\checkmark$			$\checkmark$	
Lock, Epston & Maisel (2004) [89]	$\checkmark$	$\checkmark$		$\checkmark$			$\checkmark$	
Lundby (2014) [70]	$\checkmark$		$\checkmark$	$\checkmark$	$\checkmark$		$\checkmark$	
Maisel, Epston & Borden (2004) [52]	$\checkmark$	$\checkmark$	$\checkmark$	$\checkmark$	$\checkmark$	$\checkmark$	$\checkmark$	
Nylund (2002) [91]		$\checkmark$	$\checkmark$				$\checkmark$	$\checkmark$
Pedersen (2016) [92]	$\checkmark$	$\checkmark$	$\checkmark$		$\checkmark$	$\checkmark$*	$\checkmark$	
Robbins & Pehrsson (2009) [93]		$\checkmark$	$\checkmark$		$\checkmark$		$\checkmark$	
Russell (2007) [94]	$\checkmark$			$\checkmark$		$\checkmark$	$\checkmark$	
Weber (2007) [97]		$\checkmark$		$\checkmark$			$\checkmark$	
Weber, Davis & McPhie (2006) [98]	$\checkmark$	$\checkmark$	$\checkmark$	$\checkmark$			$\checkmark$	
White (1986) [61]	$\checkmark$			$\checkmark$			$\checkmark$	
White (1991) [62]	$\checkmark$	$\checkmark$				$\checkmark$	$\checkmark$	
Zimmerman & Dickerson (1994) [99]	$\checkmark$		$\checkmark$	$\checkmark$			$\checkmark$	

*Brief reference to this component

##### Therapist stance

Seventeen articles had a focus on the key components of the therapist stance within NT. Robbins & Pehrsson [89] and Dallos [90] emphasised the importance of establishing safety, trust and security in the therapeutic relationship, particularly within the early stages of treatment. In five of the papers, power imbalances within the therapeutic relationship were explicitly named by the therapist and explored with the client during the intervention [75, 76, 82, 91, 92]. In particular, Borden [76] emphasised the importance of making visible and deconstructing discourses surrounding power dynamics within broader treatment settings, including public hospitals, community centres or private practice clinics. Six of the articles demonstrated or spoke to the process of regularly seeking feedback and asking for consent from their clients throughout narrative conversations [53, 61, 75, 92–94].

Another key component of the spirit of narrative is a collaborative therapist stance. Lainson [82] and Ingamells [92] noted instances throughout exemplar clinical transcripts where the therapist took a stance of radical genuineness and self-disclosure. Likewise, Lock, Epston & Maisel [85], Nylund [95] and Maisel, Epston & Borden [53] described the therapist stance in narrative therapy as one of curiosity and respect, rather than giving advice to the client in an authoritative manner. This collaborative approach challenges the notion that therapists hold objective knowledge in dealing with problems. Instead the narrative therapist stance supports the use of ‘co-research’ in using combined therapist and insider knowledge to develop flexible client-centred solutions [53, 61, 73, 74, 77, 87, 91, 93, 94, 96].

##### The person is the expert

Several identified articles indicated that placing the client in a position of authority and privileging their ‘insider’ knowledge is a fundamental component of the narrative worldview [73, 76, 77, 84, 96]. Nylund [95] argued for the importance of this when working with individuals with eating concerns, as they may have had previous negative, judgemental or pathologizing experiences of treatment. Lainson [82] and Pedersen [75] demonstrated attempts to ensure client consent to engage in the intervention at various points in the process. Eighteen of the articles asserted the importance of the therapeutic stance that positioned the client as an expert on their own life [75, 87, 89]. Maisel, Epston & Borden [53] positioned the person as having access to the most intimate knowledge about the problem precisely because they have lived through it. Additionally, papers by Ingamells [92], Lainson [82] and Lundby [73] suggested that viewing the person as the expert of their life was important not only in adult populations, but also when working with children, adolescents, and families. These papers emphasised the importance of the use of client-centred constructions of the problem and consideration of what recovery looks like for the individual (e.g., broadening social circles, returning to work, enjoyment of a variety of foods, etc.) [53, 78, 79, 93, 94]. Three of the articles indicated that clients were directly invited to guide the direction of therapy or contribute to the content of structured sessions, such as in group therapy programs [74, 79, 81].

##### ‘The problem is the problem’

Early work by White [59] highlighted the importance of separating the problem from the person by viewing the problem as developing despite the best efforts of the individual and their family to reach a solution. A further 14 of the articles contained reference to the importance of viewing the ‘problem as the problem’. Within this framework, the individual and/or family were not viewed as inherently inadequate or deficient, but as responding to pressures and contradictory influences within their environment. This was described in White’s [59] early work through the lens of ‘cybernetics theory’. The theory proposed that clients and families have been restrained by external forces from participating in alternative interactions and have fallen into the problem as the only workable solution to a potentially broader problem. The papers by Dallos [90], Lainson [83], Brown, Weber & Ali [78] and Brown [77] extended on this to postulate that the problem likely develops to meet an internal need, whether this be an individual/personal need (e.g., for control, to gain a sense of self-worth), outstanding attachment needs within the family, and/or to meet broader societal expectations (e.g., to be seen as thin or feminine).

##### Identity as multi-storied

Eleven of the articles spoke to a conceptualisation of a person’s identity as multi-faceted and constituted by the stories of their lives. This included how they make meaning of a range of events, lived experiences, and relationships in the social and cultural contexts of their lives. These stories shape who they understand themselves to be or their sense of self. In particular, Borden [76] argued against unitary or ‘thin’ understandings of identity, which may inadvertently reduce the person to a problem-saturated story. Similarly, Lundby [73] proposed that the primary goal of narrative family therapy is to collaboratively create multi-storied accounts of the person’s life and identity. Kraner & Ingram [81], Ibrahim & Tchanturia [93], Lainson [83], Lundby [73], Brown [77] and Lock, Epston & Maisel [85] identified that it is essential that emphasis be given to parts of the person’s identity and life that are external to the presenting problem, such as their relationships, relative strengths, hobbies, and hopes and dreams for the future. In viewing identity in this way, narrative therapy has scope to encourage an attitude of experimentation in using individual strengths to engage in anti-AN/BN acts, including creative writing, or challenging unhelpful beliefs supported by the problem [89, 92].

##### Perform the meaning

Nine papers conceptualised a person’s identity as performative and therefore lived out in everyday life. Brown, Weber & Ali [78] explored how the women communicated and performed social expectations of self-restraint through the dieting and weight control of their bodies, i.e., ‘body talk’. Likewise, other articles asserted that meaning is made through daily interactions in addition to therapy and proposed that narrative conversations explore opportunities to negotiate and perform aspects of identity [74, 76, 82, 86]. In addition to this, White [61], Pedersen [75] and Lainson [83] positioned clients’ reclaiming of life as not only cognitive, but as practical, meaningful and sustaining.

##### Language

All of the 28 studies that discussed some aspect of the narrative worldview explicitly or implicitly conveyed the importance of using language that does not pathologise, shame, place judgement or disempower the client. This linguistic shift included the practice of ‘externalisation’ of the problem. Specifically, Russell [86] and Pedersen [75] indicated that this process is not only a technique to be used within therapy, but is a way of understanding the problem and the client within a poststructuralist framework. Externalisation positions the problem in a way that challenges the over-responsibility and guilt that may be attributed to the client for an eating problem and their struggles to overcome it. Furthermore, Maisel, Epston & Borden [53] emphasised the use of ‘anti-AN/BN’ language that detaches and separates the person from the problem, so that they are able to discern their own identity and voice from the influence of the ED. This use of language was understood as being maintained by the therapist throughout the entirety of therapy, rather than an isolated and occasionally used technique. Narrative therapy was described as implicitly encouraging the client to also adopt externalising language and way of viewing themselves as distinct from the problem [87–89, 95].

##### Readiness for change

Two of the articles [74, 97] incorporated elements of the motivational interviewing intervention [98] into the intervention. In particular, they discussed the importance of working with their clients at their ‘stage of readiness to change’ [97]. This prioritises the person as having autonomy in their decisions to mobilise change in their lives. Likewise, Golan [74] concluded that the assumptions that underlie narrative therapy and motivational interviewing are able to empower the client in therapy and in their influence over the problem.

#### Unpacking of problem stories

All of the identified articles included one aspect of narrative therapy that unpacked the dominant problem story. These aspects were: the practices of deconstruction, externalisation, and/or experience-near naming.

##### Deconstruction

Thirty-one of the papers referenced the use of deconstruction of the eating problem to explore how clients had been recruited into beliefs or ideas about themselves as a person. Deconstruction was described by White [61] as the identification of taken-for-granted practices, attitudes, and ideologies that the problem story is built upon. Likewise, several articles emphasised that this process of deconstruction takes place on multiple levels, from broader social, political and cultural discourses (e.g., constructions of gender, medicalisation of EDs, the influence of media, etc.) to familial and individual factors (e.g., attachment narratives, parental scripts, etc.) [53, 74, 81, 87–89, 99, 100].

The process of deconstruction was described by many of the articles as mapping the influences the problem on the person's cognitive, affective, interpersonal, and behavioural experiences [59, 61, 74, 76, 81, 88]. For some of the articles, it was important that deconstruction was undertaken not only for the problem and its meaning to the person, but in regard to their experiences of psychological treatment. This included the authority that is often given to health professionals in defining sickness and wellness [76, 81, 88, 91]. Additionally, Epston, Morris & Maisel [100] and Zimmerman & Dickerson [88] considered how resources from others who share a lived ED experience (‘Th/e Anti-AN/BN League’) could be drawn on in the process of deconstruction. This was done through individuals reading the stories of others who shared similar experiences, including societal influences that may have recruited them into a problematic relationship with eating and their body.

##### Externalisation

Thirty of the articles included reference to the practice of externalisation in unpacking the problem story. Lock, Epston, Maisel & de Faria [84] explained externalisation as the positioning of the problem outside of the person, meaning that it can then be objectified and a cognitive stance taken with respect to it. Many of the studies used externalisation with the person’s language forms, including the use of metaphor and personification of the eating problem [59, 74, 76–83, 88, 90, 92–94, 97, 99, 101–103]. Using externalising language to separate the client from the problem allowed for the tactics, intentions and agendas of the problem to be explored, assisting the person to engage critically in deconstructing the problem [53, 73, 85, 86, 89, 92, 100]. Some of the studies also utilised externalisation in creative writing formats, such as writing poems to personify the problem and short text passages about the negative impacts of the problem [85, 89, 95]. Likewise, Weber, Davis & McPhie [87], Nylund [95] and Lock, Epston & Maisel [85] used roleplaying exercises to highlight differences between the client’s voice and that of the problem. Roleplays were also used to challenge the problem directly and encourage perspective taking (e.g., family members role-playing each other to explore their varying thoughts, feelings and experiences of a situation).

##### Experience-near naming

Twenty-one of the 33 articles referenced the importance of using client’s own words and phrases to explore the problem and its effects, rather than using medical discourse, such as ‘eating disorder’, ‘anorexia’ or ‘bulimia’ nervosa. This was achieved by asking clients which of the available ways to speak about the problem they preferred [75, 94] and the therapist using the client’s own words to refer to the identified problem [53, 61, 73, 79, 81–83, 86–88, 93, 101, 102]. Maisel, Epston & Borden [53] provided several examples of this, including the ‘dark tunnel’ (p. 65), ‘devil’ (p. 63), ‘evil’ (p. 92), and ‘death row’ (p. 107). These examples highlight the range of metaphors use (for example ‘dark tunnel’ is a geographical metaphor, whereas ‘evil’ is an adversarial metaphor) for their experiences. The papers outlined the experience-near metaphors that therapists took up in therapeutic conversations. Three of the articles also spoke about the importance of using the client’s words to name and thicken the hidden story once generated in therapeutic conversations, e.g., ‘caring for the self’ [102], ‘big step’ and ‘small miracle’ [73], and ‘getting well’ [53].

#### Finding hidden openings

##### Identification of hidden openings or unique outcomes

Twenty-eight of the articles outlined ways that hidden openings or ‘unique outcomes’ were explored. This included the practice of ‘double listening’ [61], whereby the clinician explored the impacts the problem has had on the person’s life, and noticed any unique outcomes that did not fit with the dominant problem story [59, 73, 74, 86, 99, 101]. Identification of unique outcomes was achieved in a variety of ways and involved exploring past, current, and possible future acts of resistance against the problem or anti-problem actions, thoughts, desires and attitudes [61, 78, 80, 85, 89, 92, 95, 100]. Four of the studies extended this idea to suggest that any action that lay outside of stories built on socially or culturally constructed taken for granted assumptions could be understood as an act of political resistance or unrestrained expression of the self [53, 75, 83, 86].

##### Exploring ways of living out of sync with the problem story

For some of the articles, unique outcomes were generated through ‘scaffolding conversations’. These conversations noticed and re-authored instances where the person had escaped the influence of the problem [102]. In this process, new identity stories were generated for old experiences. These new-old stories [64] were thickened through linking these previously un-authored actions with the client’s values and personal aptitudes [74, 77, 79, 81] and the development of counter-narratives that stood in opposition to the problem narrative [59, 85]. Several of the articles incorporated naming the aspects of a client’s experience and identity that had previously been left ‘unstoried’ [53, 73, 103].

##### Absent but implicit

Five of the articles explored looking for the ‘absent but implicit’ [63] to identify hidden openings. The ‘absent but implicit’ was described by the papers as engaging the person to explore what was implicit and relied upon in their discernment of their experiences as problematic [61, 76, 77, 96, 100, 103]. For example, Howells [101] used the metaphor of ‘home’ to explore what was currently absent from the client’s conceptualisation of home that they valued. Additionally, Tsun on-Kee [103] explored what was absent but implicit in the client’s experience of guilt about their eating patterns. This revealed openings to previously hidden stories based on intentions, beliefs and hopes for the future (for example, food being culturally important and family-centred, and the need for self-forgiveness).

#### Thickening the meaning of hidden and revealed stories

Thirty of the articles included reference to methods of thickening and living out the hidden stories, once their openings had been revealed. The process of thickening hidden stories was described by the papers as building a detailed picture of what life outside of the problem looked like. This was done through developing and naming counter-narratives that more flexibly described the person’s experience [61, 74, 79, 85, 86, 90, 101–103]. These alternate storylines were built and strengthened by expanding on unique outcomes to explore thoughts, feelings or actions outside of the problem story in the past, present and future [73, 80].

Likewise, several papers noted that hidden stories could be further thickened by exploring what specific acts of resistance may have to say about the individual’s values, beliefs, identity or political ideals [53, 75, 76, 79, 86, 101]. Epston, Morris & Maisel [100] described the thickening process as establishing a ‘history’ of the values underlying anti-AN/BN achievements by exploring other instances in a person’s life where they stood against AN/BN. This in turn further strengthened the once obscured narrative. For Lainson [83], the process of thickening hidden stories and identities was centred on individuals taking up new priorities that were consistent with their values. These value-consistent values were cultivated so that they occupied more space in the person’s life than the ED. Ingamells [92] explored future implications and potential actions if the person with lived experience were to ‘live out’ their new identity (e.g., ‘Are you about to become Wilbur the Warrior?’ and ‘Is there a little bit more of warrior in Wilbur than there was the last time we met?’). The papers cited examples of other ways of thickening hidden stories, including letter writing and creating an audience to witness the stories previously obscured by the problem narrative.

##### Letter and creative writing

Twelve articles included the use of narrative letter writing to thicken and strengthen client’s hidden storylines between therapy sessions and at the conclusion of treatment. Many articles referenced the use of therapist written letters to the client following sessions. These letters included prompting questions and reflections to be considered at the beginning of the next session [53, 73, 75, 79, 89, 95, 97, 100, 101]. Some of the articles utilised creative or poetry writing between sessions to encourage acts of resistance against the problem or to thicken the client’s once obscured identity [53, 75, 95, 100]. In particular, Lainson [82] and Nylund [95] utilised creative writing processes to highlight individual’s behaviours and activities that stood as protests against the problem story. Other articles used structured writing as a means of farewelling clients and concluding regular sessions, whilst providing them with a reminder of their developments and achievements throughout treatment [74, 75, 79, 93].

##### Outsider witnessing

Many of the identified studies referenced the use of outsider witnessing to thicken hidden stories. This process involved inviting an audience of observers of the client’s journey to act as witnesses to the ways in which they had seen the client living out the hidden and revealed storylines in the present and in the past [53]. Seven of the articles utilised close family members and friends and/or members of the therapeutic group to witness counter-narratives to the problem story. This included how the person’s actions spoke to their underlying values or identities and acts of resistance they had witnessed the individual take against the problem [61, 75, 76, 80, 81, 85, 93]. Other articles included the use of rituals or celebratory ceremonies with loved ones where individuals celebrated their newly revised relationship with the problem and with themselves [74, 79, 95, 101, 103]. Two papers noted that they provided clients with a physical certificate or memorabilia to stand as tangible evidence of their recovery at these ceremonies [82, 100]. Likewise, an additional six articles encouraged individuals to produce creative writing or works of poetry that could be contributed to ‘The Anti-AN/BN League’ or circulated within communities of others with ED problems [82, 85, 87, 89, 95, 100].

##### Safety considerations

Six out of the total 33 articles mentioned the importance of establishing safety when working with indivudals with a lived ED experience and outlined how this could be integrated into a narrative therapy framework. Two of these papers reported a process of initial assessment screening and exclusion criteria to determine any clients for which the intervention may not be adequate or appropriate at the time of the assessment [80, 87]. This included women who had multiple medical problems or life-threatening eating-disturbed behaviour, those who were assessed as having higher or different needs, and/or those who had a recent history of a previous suicide attempt. Likewise, 3 of these papers employed a multidisciplinary approach to allow physical and mental health professionals to work together to ensure medical and psychological safety for the clients [74, 94, 102]. Such teams were comprised of referring general practitioners, nutritionists, dietitians, nursing staff, and psychiatrists. Specifically, Scott, Hanstock & Patterson-Kane [102] and Golan [74] noted that client engagement with nutritional counselling and regular medical reviews were vital components of the treatment program [74, 102].

Maisel, Epston & Borden [53] extensively discussed how essential medical practices may be understood and utilised whilst still maintaining a narrative worldview. In particular, the use of inpatient practices that may be imposing or coercive were considered. They asserted that whilst impositions may be needed at times to save lives, there are precautions that can be taken ahead of time to mitigate the damage caused by the experience. This included gaining information about the client’s wishes should they require tube-feeding or other invasive procedures and obtaining a kind of ‘pre-consent’.

## Discussion

This narrative synthesis has grouped and mapped the following components across the selected studies: (A) the narrative worldview, (B) unpacking the problem story, (C) finding hidden openings, (D) thickening the meaning of hidden and revealed stories, and (E) safety considerations.

The findings of this synthesis indicated that the underlying philosophies and frameworks of narrative therapy (i.e., ‘the narrative worldview’) were evident and well-embedded in the in the delivery of narrative interventions and practice of subsequent techniques across the selected studies. In other words, “the how” of narrative therapy was emphasised in addition to “the what” of the practice components of narrative therapy. Within the articles, this worldview was located within the philosophical traditions of social constructionism and poststructuralism [52], including an emphasis of therapy unpacking or deconstructing social, cultural and political ideas and expectations [60, 65]. The spirit of narrative therapy was evident across the articles, including the view that the person is: (1) not the problem, the problem is the problem, and (2) the expert of their life. Many articles discussed the importance of the therapist taking a collaborative, genuine, and curious stance. Despite this, few of the articles discussed the importance of therapists working with a client’s readiness to change [97].

In relation to therapy content, all of the articles mentioned the process of unpacking dominant problem stories, including deconstruction of dominant societal ideas and expectations and mapping the influence of the problem on a person’s life [53, 60, 65]. The way in which externalisation [52, 66] was described and used across the articles was of particular interest, with this narrative practice being the most frequently cited and exemplified. Many of the papers referenced the use of linguistic shifts and role-playing to externalise and unpack the problem story, however, this was infrequently linked to the overall narrative worldview where the person is positioned as the expert of their life. For some papers, externalisation was linked to the non-pathologising narrative approach and use of ‘experience-near naming’ [37, 52, 56, 58, 66, 104].

However, the majority of the articles spoke of externalisation as a therapeutic technique rather than positioning it within the broader narrative worldview. Externalisation as a technique in interventions for EDs is not limited to narrative therapy. For example, “externalisation of the illness” has been integrated as a therapeutic practice in Maudsley Family Therapy, FBT [18, 105] and CBT-AN [11]. The uniqueness of narrative therapy appeared not from the use of specific techniques, such as externalisation, but because of the broader spirit or worldview that informs narrative therapy practices. This includes the use of experience near naming, where the client is positioned as the expert on how the problem is talked about (e.g. as a “dark tunnel” rather than through the medical language of “illness”).

Similarly, the majority of the articles discussed the process of identification of “unique outcomes” [60] to explore hidden stories by using techniques such as “double listening” [61], analysis of “acts of resistance” and “scaffolding conversations” [59, 102]. Despite this, few papers spoke about narrative therapy’s conceptualisation of a person’s identity as multi-storied [60, 106] and the importance of focusing on aspects of the clients’ values, hopes and strengths that have not been taken over by the problem [59, 62]. The papers emphasised techniques and methods of cognitively thickening hidden stories using counter-narratives and re-authoring conversations [54, 69]. There was a noticeable lack of exploration and explanation of how identity shifts that occurred in therapy (for example, as evident in transcripts of therapy sessions) were then performed or lived out in the person’s everyday life [106]. Therefore, there was frequently little indication about whether clients cited in the papers performed the meaning of these identity shifts, including whether there was a corresponding improvement in their eating patterns and relationship with their bodies.

Sackett [107] has purported that evidence-based practice consists of client preferences, clinician experiences, and research outcomes. Some of the papers spoke to the clinician’s experience and client preferences for treatment, including how the client’s expertise was honoured in their life, and the clinician’s experience in tailoring treatment to the client. Despite this, a relatively low number of the papers incorporated a future focus on what the client’s life might look like (e.g., their thoughts, feelings, and actions) if they were to live out the identity shifts that were more comprehensively noted in the papers. This included a lack of discussion of meaningful treatment outcomes in relation to ED symptoms in most papers, conceptualised in narrative therapy as the extent to which the person performed the meaning of the identity shifts noted in therapy. Importantly, few of the papers included information regarding how therapists established safety within the narrative treatment framework. Some of the articles included safety-related exclusion criteria for narrative therapy interventions, however, even fewer explicitly mentioned practices whereby safety was assessed and established – for example, ongoing review of eating patterns and body weight. There were also few papers that raised the importance of a multidisciplinary team when working with EDs and how this fits within the narrative framework.

### Limitations and strengths

Through synthesising and analysing the data from included articles, researcher judgements were made regarding the quality and contribution of each of the papers. The most frequently observed limitation in the overall quality of the papers was a lack of evidence for ethics approval or considerations for ethical practice. Additionally, the majority of articles had a small number of participants (often in a case study design format) and only half of the papers mentioned the ways that identity shifts were performed. This included a lack of focus on ways that the reported narrative interventions impacted an individual’s eating patterns and their relationship with their body. Furthermore, many of the papers were written by narrative therapists who were delivering the treatment and reflecting on the components used. This indicates that there may be a risk of bias present in the papers regarding selection and performance/confounding bias.

Despite the limitations associated with both this paper and the articles it studied, there are a number of strengths associated with this review. Specifically, this paper was developed in response to the current paucity of research synthesising narrative therapy interventions for EDs thus far. Additionally, this synthesis incorporates the findings from a variety of articles across different treatment settings and contexts and provides a synthesis of the current publications on narrative therapy for EDs. In doing so, it has illuminated the core components of narrative therapy for EDs and provides potential foci for future research.

### Implications

The findings of this review demonstrated that the current literature on narrative therapy for EDs emphasises not only the use of specific therapeutic techniques but also the *process* of narrative therapy. This therapeutic process is informed by the narrative worldview where the person is (1) positioned as the expert of their life; and (2) not the problem but rather the problem is the problem. The selected papers provide a particular sort of evidence that is, the first-hand experience of narrative practitioners who report narrative therapy to be a helpful way to engage clients in the treatment of ED. This has been termed by David Epston as practice-based evidence [108], which is one arm evidence-based practice informed by therapist expertise [109].

The results of this synthesis emphasise the need for further research into the processes of identity shifts in narrative therapy, as performed by the person with a lived experience. This includes how they are engaging in their life, relationships, eating patterns/behaviours and their relationship with their body. This will include clinicians and researchers developing ways to observe and systematically measure of eating pattern/behaviour changes to explore the proposed inter-relationship between identity and its performance. A dilemma of researching narrative therapy has been outlined by Epston, Stillman & Erbes [109] as: “…science is about generalising broad truths that apply to everyone, while narrative is about elucidating local truths that apply to those who construct them and live them” (p. 77). Therefore, a challenge for researchers and clinicians is how to make observations and measurements in a way that aligns with the spirit of narrative therapy, which includes a non-interrogative approach, a prioritisation of the voice of the experiencing person, and maintaining the position that they are the expert of their life in this assessment [109].

Furthermore, this synthesis has identified that there are considerable gaps within our current understandings of how issues pertaining to client safety should be explored within the narrative worldview [1]. This is important given the significant and sometimes severe medical complications and life-saving nature of treatment associated with working with individuals experiencing EDs, including within multidisciplinary teams. It is vital that papers document ways that narrative therapy interventions for EDs more explicitly address safety considerations, whilst maintaining the spirit and worldview of intervention, including client respect and autonomy, positioning them as the expert of their life and the problem as the problem (not the person). In other words, what does safety look like ‘the narrative way?’.

### Concluding remarks

The exploration of the nature and effectiveness of narrative therapy for the treatment of EDs is an emerging area of research. The findings of this systematic review further the understanding of the specific components of narrative therapy that are currently well documented and used in clinical practice in the treatment of EDs. Additional research is needed to further this understanding of narrative therapy in the field of EDs and how this intervention facilitates recovery of life and identity from an ED. These approaches have scope to extend the conversation about narrative therapy for EDs to more comprehensively: (1) address how safety considerations are integrated into the narrative framework and with the spirit of narrative therapy, (2) employ co-design research methodologies that include the experiences and perspectives of people with lived experience, and (3) address ways that key identity shifts are performed or lived out in a client’s life and the person-centred assessment of these, so that meaningful change becomes evidenced beyond the therapy room.

### Supplementary Information

**Additional file 1.** Study quality and Narrative Therapy components.

## Data Availability

Not Appilicable.

## Appendix

**Table:**

List of abbreviations
ED: Eating Disorder
AN: Anorexia Nervosa
BN: Bulimia Nervosa
BED: Binge eating Disorder
CBT: Cognitive Behavioural Therapy
FBT: Family-Based Therapy
