# Supplementary material for: Paper 1: a systematic synthesis of narrative therapy treatment components for the treatment of eating disorders
Source: J Eat Disord. 2022 Sep 8;10:137. doi: 10.1186/s40337-022-00635-5 (PMC9461296; doi:10.1186/s40337-022-00635-5)
Supplement: Supplementary file 1 — Additional file 1. Study quality and Narrative Therapy components. [file 40337_2022_635_MOESM1_ESM.docx]

**ADDITIONAL Files: Narrative Therapy Systematic Review – Therapy Components and Outcomes**Table A: Quality Appraisal Ratings for Articles

| **Author/s (Year)** | **1. Reference to Extant Literature** | **2. Hypothesis/aim/ objective described** | **3. Main outcomes described** | **4. Sample adequately described** | **5. Representative sample size** | **6. Components of intervention described** | **7. Main findings described** | **8. Logical conclusion/position** | **9. Any incongruence with pre-existing literature logically defended** | **10. Evidence of approval/practice** |
| --- | --- | --- | --- | --- | --- | --- | --- | --- | --- | --- |
| Beaudoin (2020) (1) | 2 | 1 | 1 | 2 | 1 | 2 | 2 | 2 | 2 | 1 |
| Borden (2007) (2) | 2 | 2 | 1 | 0 | 1 | 2 | 1 | 2 | 2 | 1 |
| Brown (2018) (3) | 2 | 2 | 1 | 2 | 1 | 2 | 1 | 1 | 1 | 1 |
| Brown, Weber & Ali (2008) (4) | 1 | 1 | 0 | 1 | 1 | 2 | 2 | 2 | 1 | 1 |
| Courtney & Williams (2000) (5) | 1 | 1 | 0 | 1 | 1 | 2 | 2 | 2 | 1 | 1 |
| Craggs & Reed (2007) (6) | 2 | 2 | 1 | 2 | 1 | 2 | 2 | 2 | 2 | 1 |
| Dallos (2004) (7) | 2 | 2 | 1 | 2 | 1 | 2 | 1 | 2 | 2 | 1 |
| Davidson & Birmingham (2001) (8) | 2 | 2 | 1 | 2 | 1 | 2 | 2 | 1 | 0 | 1 |
| Dennstedt (2010) (9) | 2 | 2 | 1 | 1 | 1 | 1 | 1 | 2 | 2 | 1 |
| Epston, Morris & Maisel (1995) (10) | 2 | 1 | 1 | 1 | 1 | 2 | 1 | 1 | 1 | 1 |
| Golan (2013) (11) | 2 | 2 | 2 | 2 | 2 | 2 | 2 | 2 | 1 | 1 |
| Howells (2009) (12) | 1 | 2 | 1 | 1 | 1 | 2 | 1 | 2 | 1 | 1 |
| Ibhrahim & Tchanturia (2018) (13) | 2 | 2 | 2 | 2 | 2 | 2 | 2 | 2 | 2 | 1 |
| Ingamells (2016) (14) | 0 | 2 | 2 | 2 | 0 | 2 | 1 | 1 | 0 | 1 |
| Kantor (2000) (15) | 2 | 2 | 2 | 2 | 2 | 2 | 2 | 2 | 2 | 2 |
| Kraner & Ingram (1997) (16) | 2 | 2 | 2 | 1 | 2 | 2 | 2 | 2 | 1 | 1 |
| Lainson (2016) (17) | 2 | 2 | 1 | 2 | 1 | 2 | 1 | 2 | 2 | 1 |
| Lainson (2019) (18) | 2 | 2 | 1 | 1 | 1 | 2 | 1 | 2 | 2 | 1 |
| Lock, Epston & Maisel (2004) (19) | 2 | 0 | 0 | 0 | 1 | 2 | 2 | 2 | 2 | 1 |
| Lock, Epston, Maisel & de Faria (2005) (20) | 2 | 1 | 0 | 0 | 0 | 2 | 0 | 2 | 2 | 1 |
| Lundby (2014) (21) | 2 | 2 | 1 | 2 | 1 | 2 | 1 | 2 | 1 | 1 |
| Nylund (2002) (22) | 2 | 2 | 1 | 2 | 1 | 2 | 1 | 2 | 2 | 1 |
| Pedersen (2016) (23) | 1 | 2 | 0 | 2 | 1 | 2 | 1 | 1 | 1 | 1 |
| Robbins & Pehrsson (2009) (24) | 2 | 2 | 1 | 2 | 1 | 2 | 1 | 2 | 2 | 1 |
| Russell (2007) (25) | 0 | 1 | 0 | 2 | 1 | 2 | 1 | 1 | 0 | 1 |
| Scott, Hanstock & Patterson-Kane (2013) (26) | 2 | 2 | 2 | 2 | 1 | 2 | 2 | 2 | 2 | 1 |
| Tsun on-Kee (2011) (27) | 2 | 2 | 0 | 2 | 1 | 2 | 2 | 1 | 1 | 1 |
| Weber (2007) (28) | 2 | 2 | 1 | 0 | 1 | 2 | 2 | 2 | 1 | 1 |
| Weber, Davis & McPhie (2006) (29) | 2 | 2 | 2 | 2 | 2 | 2 | 2 | 2 | 2 | 2 |
| White (1986) (30) | 2 | 2 | 0 | 2 | 1 | 2 | 1 | 2 | 2 | 1 |
| White (1991) (31) | 2 | 2 | 1 | 2 | 1 | 2 | 1 | 2 | 2 | 1 |
| Zimmerman & Dickerson (1994) (32) | 2 | 2 | 1 | 2 | 1 | 2 | 1 | 2 | 1 | 1 |

*Where 0 = No, 1 = Unclear, and 2 = Yes.*

Table B: Quality Appraisal of Book

| **Author/s (Year)** | **1. Source of opinion clearly identified** | **2. Source of opinion has standing in the field of expertise** | **3. Interests of the relevant population are the central focus of the opinion** | **4. Logical conclusions/position** | **5. Reference to extant literature** | **6. Any incongruence with the literature/sources is logically defended** |
| --- | --- | --- | --- | --- | --- | --- |
| Maisel, Epston & Borden (2004) (33) | 2 | 2 | 2 | 2 | 2 | 1 |

*Where 0 = No, 1 = Unclear, and 2 = Yes.*

Table C: Data Extraction Summary of narrative therapy treatment interventions and outcomes

| **Author/s (Year)** | **Aims** | **Sample Characteristics** | **Treatment Details** | **Outcomes** |
| --- | --- | --- | --- | --- |
| Beaudoin (2020) (1) | To expand on the concept of double listening by incorporating affective experience | N: 1 (Case Study)   - ‘Edith’ (female, age unknown, college student who struggled with episodes of binge-eating)   Country: USA  Language: English | Narrative therapy  Individual sessions (number unknown)  Treatment context unknown | Qualitative data (as reported by the client):   - Finished intervention with ‘hope’, ‘new understandings’, ‘less confusion’, ‘perspective’, ‘feelings of being capable’, and able to ‘discern the different sensations in her body’ - Felt more in control of ED symptoms, increased self-compassion, preferred identity was thickened   No quantitative data provided |
| Borden (2007) (2) | Seeks to describe strategies used in group therapeutic settings to negotiate meaning and perform identity, by taking apart assumptions about eating problems and recovery | Limited details regarding clients that participated in the group program – clients all had eating and/or weight related concerns  Exerts are provided from four female clients who completed the program - ‘Lara’, ‘Nikki’, Katie’ and ‘Anne’ (ages unknown)  Country: USA  Language: English | Narrative therapy  Group program format, with initial individual or family session upon admission  Seen in day treatment program at ED specific treatment centre (requires financial support from family members or insurance company) | Qualitative data (as reported by clients):   - Increased trust in self, setting of appropriate limits and boundaries, prioritising self-care and own needs, and developing self-compassion   No quantitative data provided |
| Brown (2018) (3) | Explores how feminist narrative practice provides a framework for working with clients with trauma backgrounds | N: 1 (Case study)   - ‘Zelda’ (female, 26, history of complex trauma, presenting with depression and binging and purging behaviours)   Country: Canada  Language: English | Narrative therapy  Individual sessions (number unknown)  Seen in private practice setting | Qualitative data (as reported by the client):   - Increased ability to talk about abuse directly and indirectly, to reflect on experiences and coping, and to challenge ideas of self-blame, worthlessness and assumptions about others - Emergence of the hidden story allowed the client to acknowledge that the abuse was oppressive and wrong - Ability to acknowledge the importance of emotional needs, self-care and self-compassion   No quantitative data provided |
| Brown, Weber & Ali (2008) (4) | Proposes an alternative treatment approach for EDs using a combination of feminist and narrative therapies | N:1 (Case Study)   - ‘Shayna’ (female, 23, episodic BN that commenced at age 16)   Country: Canada  Language: English | Narrative therapy  Individual sessions for approximately 18 months  Seen in private practice setting | Qualitative data (as reported by the client):   - The client had a greater understanding of the meanings they associated with BN, which allowed dominant discourses to be challenged - Increased self-expression, stable body weight, limited/decreased binging and purging episodes   No quantitative data provided |
| Courtney & Williams (2000) (5) | Proposes group-based intervention for body image and eating issues using a narrative framework – i.e., ‘scaffolding’ of narrative ideas, rather than structured written courses/manual | Limited details regarding specific clients  Group based intervention typically including:   - Between four to six women - Ranging in age from early twenties to mid-fifties - Anglo-Australian clients   Country: Australia  Language: English | Narrative therapy  Eight sessions of two-hourly group meetings held weekly  Seen in community health centre setting | Qualitative data (as reported by the client/s):   - Clients reported that speaking with other women about eating issues in a supportive, respectful and validating environments was valuable, including being an audience to other women’s stories - Clients indicated they felt a shift in the relationship they held with their eating problem   No quantitative data provided |
| Craggs & Reed (2007) (6) | Co-authored article by therapist and client to explore the therapeutic process from different vantage points, with emphasis on the influence of the clinical context | N: 2 (Client and therapist provided reflections on their experiences of treatment)   - Client: ‘Tracy’ (female, early adulthood, employed as researcher) - Therapist: ‘Alex’ (male, middle-aged, family therapist)   Country: England  Language: English | Narrative approach  Process of inquiry resembled the ‘co-operative inquiry’/’co-research’ approach (Heron, 1996)  Seen in a specialist ED service in a general hospital | Qualitative data (as indicated by reflections of therapist and service-user):   - Client’s commentary suggested uneasiness and frustration with traditional therapy contexts (i.e., hospitals) and difficulties with interpersonal communication - Client identified their own speaking positions which they may not have been able to do otherwise - Therapist’s reflection indicating that reading the client’s account had assisted them to realise more fully the influence of the professional agency context which could be a source of anxiety and stress for clients   No quantitative data provided |
| Dallos (2004) (7) | Considers contributions that attachment and narrative therapies can offer to the treatment of EDs | N: 1 (Case Study)   - ‘Mary’ (female, 19, diagnosis of AN, recent inpatient admission for three months and outpatient day-program contact for a further six months)   Country: England  Language: English | Narrative therapy  Combination of family therapy sessions (eight in total), monthly sessions with Mary’s parents (number unknown), and occasional six-weekly individual sessions with Mary (number unknown)  Seen in private practice setting | Qualitative data (as reported by client and her family):   - Client seemed to be less triangulated between her parents, and increased emotional independence - Client was better able to spend time with her friends, return to work, and avoid readmission to an inpatient unit   No quantitative data provided |
| Davidson & Birmingham (2001) (8) | To review the recently pioneered processes and use of Therapeutic Letter Writing (TLW) in individuals with eating concerns | N: 1 (Case Study)   - ‘Jane’ (female, age unknown, seen for eating concerns and family difficulties)   Country: Canada  Language: English | Narrative therapy and motivational interviewing, with focus on TLR  Individual and family sessions (letters written following sessions with family therapist)  Seen in outpatient day program at a hospital | Qualitative data (as reported by client and reflections from therapist):   - TLW makes misunderstandings more evident and functions as tangible evidence for therapist’s respect for the client - Assists therapist in reflecting and integrating ideas - Promotes transparency and respectful documentation - TLR can assist in resolving emotional separation with parents and empowering clients who may find themselves in ‘peacemaker’ role within the family unit - Distant relative became more involved and promoted emotional closeness by providing a framework for discussion of how parenting practices may have contributed to presenting difficulties - Increased honesty and improved relationships between clients and parents - In follow-up contact (approximately 2-3 months later), client described having an excellent relationship with her parents and credited TLR as being the catalyst for this   No quantitative data was provided |
| Dennstedt (2010) (9) | Explored the similarities and interplays of EDs and substance use and discussed potential implications for therapeutic intervention | Limited details regarding clients   - Case examples from seven semi-structured interviews with young women who self-identified as having lived experience of EDs and substance misuse (e.g., ‘Hannah’, ‘Beth’ ‘Ida’, ‘Ava’, ‘Jill’) - No other client details provided   Country: Canada  Language: English | Narrative therapy  Individual sessions (number unknown)  Seen in private practice setting | Qualitative data (as reported by the client/s):   - Found it helpful for both substance misuse and eating problems to be discussed, as well as how the problems interact and are related - Questions that centred on what the client values and how they make meaning of their life were regarded as an important aspect of recovery   No quantitative data provided |
| Epston, Morris & Maisel (1995) (10) | Provides details of the use of narrative therapy for AN/BN in the context of revised correspondence between the first two authors | N: 7 – Collection of case studies and exerts from clients, including:   - ‘Julie’ (female, 30) - ‘Rhonda’ (female, 19) - ‘Bridget’ (female, 15) - Rebecca (female, age unknown) - ‘Rosemarie’ (female, 23) - ‘Jane’ (female, 22) - ‘Fran’ – the most detailed case (female, age unknown, second author, 23-year lived experience of AN/BN)   Country: Unspecified – ‘Fran’ (the most detailed case study) was seen in New Zealand  Language: English | Narrative therapy  Indication that Fran was self-referred to private practice setting, and was seen for five sessions  No further details of other case studies reported | Qualitative data (as reported by client/s and indicated in exerts):   - Stories of empowerment and liberation of others with lived ED experience can be of great benefit to clients - Ms Morris was able to ‘reclaim’ her life – moving overseas, selling her house, and taking up hobbies - Increased self-confidence and self-efficacy - Engaging in self-care and valuing of her own needs and desires - Discovery of a new kind of relationship with the self - Highlighted the importance of the political slant and the effect of dominant discourses around AN/BN - Recovery conceptualised not as the absence of AN but as a change in the relationship the client has with their eating concerns   No quantitative data provided |
| Golan (2013) (11) | Outlines the processes used by multidisciplinary team in treatment of severe and enduring eating concerns  Utilises integrative model of narrative and motivational interviewing described using a five-phase journey | N: 645 (387 BN and 258 AN)   - Age range: 11-40 - Duration of illness: approximately 6-7 years   Country: Israel  Language: Hebrew | Narrative counselling and motivational interviewing  Seen in community-based facility  Delivered in context of multidisciplinary team model including family therapy, nutritional counselling, psychiatric care, individual sessions, clinical mentor | Quantitative data:   - Fewer than 10% of clients dropped out during first two months of treatment (<12% BN & <8% AN). - Treatment outcomes assessed using a Global Clinical Score based on the Average Outcome Score (Garfinkel, Moldosky & Garner, 1979) - At end of treatment, 69% of AN clients and 81% of BN clients were in a fully recovered or much improved state (i.e., full recovery defined as having been in full remission more than 12 months, and much improved defined as partial remission with infrequent occurrence of symptoms). - Four-year post treatment follow-up: 68% of AN clients and 83% of BN clients were either fully recovered or much improved   All clients who completed the program went on to secure employment |
| Howells (2009) (12) | Explores how ‘homes’ (both physical and metaphorical) can be taken up in narrative therapy | N: 3 (Case Studies)   - ‘Sally’ and ‘Tom’ (couple who were seen for marriage counselling, ages unknown) - ‘Iona’ (female, 21, seen for eating concerns) - ‘Grace’ (female, age unknown, seen for relationship difficulties)   Country: Singapore  Language: English | Narrative therapy  Individual and couple sessions (number unknown)  Seen in private practice setting | Qualitative data (as reported by client and reflections from therapist):   - Metaphor of ‘home’ was useful in envisioning and performing the meaning of a preferred future by exploring client values and providing concrete points of reference - Idea of ‘home’ assisted in separating the problem from the client/s and mapping relative influence - Clients were better equipped to establish firm boundaries in their relationships   No quantitative data provided |
| Ibhrahim & Tchanturia (2018) (13) | Exploration of how the Tree of Life (ToL) therapeutic tool can be used in the context of a narrative group intervention for women with eating concerns and depression | N: 4 (Group therapy program)   - All females - Aged 18-30 - Diagnosis of AN   Country: England  Language: English | Narrative therapy with emphasis on TOL tool  Group therapy intervention (eight weekly sessions, two facilitators, sessions were one hour in length)  Clients seen as day patients at a specialist ED service | Qualitative thematic analysis was used to summarise participants’ reflections of the group:   - Creative and visual elements helped clients remember what they learned - Clients indicated they found it helpful to focus on something other than the ED, and expressed the group offered an alternate perspective - The ToL helped clients to generate dynamic and flexible hopes for the future - Clients felt more connected and supportive of each other rather than feeling ‘in competition’   No quantitative data provided |
| Ingamells (2016) (14) | Illustrates the process of using narrative practices in family therapy for a young boy struggling with AN and anxiety | N:1 (Case Study)   - ‘Wilbur’ (male, 8, seen for AN and anxiety, illness duration of three-years)   Country: New Zealand  Language: English | Narrative therapy  Four weekly family sessions with Wilbur and his parents  Seen in private practice setting | Qualitative data (as reported by the parents of the client):   - Increased weight gain, positive thinking, less preoccupation with weight and appearance, general improvement in mood - Increased confidence, improved self-esteem, more readily trying new things, letting parents take photos of him (i.e., less concerned about social judgement)   No quantitative data provided |
| Kantor (2000) (14) | Exploration of the use of narrative therapy with individual adolescent females with eating disturbed behaviour | N: 8 (Individual)   - All females - Aged 12-17 - Selected from waiting list of potential ED clients for the psychiatry outpatient unit - Eating related concerns and/or restrictive/excessive exercise behaviour | Narrative therapy  Individual sessions  Seen in the context of a counselling centre for children and adolescents | Qualitative data:   - Client themes: control, self-criticism, negative attitude toward school, parent-teen conflict, grief/loss, body image, questionable therapy commitment. - Analysis of practice: construction of the problem, externalisation, collaboration with client, perspectives, temporal issues, meaning making, and therapist situation. - Progress for all clients with respect to reduction in the problem foci they had chosen to address during first sessions - Positive change seemed related to the number of sessions attended - Client progress included improvement in peer relationships, relationships with mothers, mood, school issues, and overall problem focus. |
| Kraner & Ingram (1997) (16) | Description of the process of establishing a group program for young women diagnosed with AN  Exploration of strategies used to promote opportunities for reflection and identification of key outcomes | N: 5 (Group therapy program)   - All females - Aged 14-17 - Diagnosis of AN - Recently discharged from inpatient hospitalisation for eating concerns   Country: Australia  Language: English | Narrative therapy  Group program (weekly meetings for eleven weeks)  Seen in the context of an outpatient child and adolescent psychiatry service attached to a metropolitan public hospital | Qualitative data (as reported by clients and identified from extracts):   - Increased self-esteem and ability to communicate assertively - Longer periods of time spent out of hospital, feelings of hopefulness, and greater connection with other group members - Increased ability to socialise with friends, and improved family relationships   No quantitative data provided |
| Lainson (2016) (17) | Exploration of how narrative conversations can assist in using both individual experiences and collective considerations  Reflective of a broader sample of clients who are regarded as ‘high functioning’ and ‘successful’, yet experience mood and eating concerns | N: 2 (Case Studies)   - ‘Ruby’ (female, 17, seen for depression and body image concerns) - ‘Natalia’ (female, 15, recently discharged from public health service, diagnosis of AN)   Country: New Zealand  Language: English | Narrative therapy  Individual sessions (number unknown)  Seen in small community counselling service | Qualitative data (as reported by the client/s and reflections from therapist):   - Narrative conversations can be used to pique intellectual curiosity and political action can precede or be the trajectory for recovery - Increased traction in sessions - Reclaiming of dignity – i.e., rejection of notions of a ‘faulty self’ - Increased engagement in and enjoyment of the therapy conversations   No quantitative data was provided |
| Lainson (2019) (18) | Exploration of how neuroscience and narrative therapy construct differing versions of the AN experience  Highlights potential problematic views perpetuated by the brain-based model of AN and argues for the privileging of insider knowledge | N: 1 (Case Study)   - ‘Laura’ (female, 30, mother to a growing family, diagnosed with AN in adolescence, presenting with ongoing eating concerns)   Country: Australia  Language: English | Narrative therapy  Individual sessions (number unknown)  Seen in private practice setting | Qualitative data (as reported by the client):   - Conversations were helpful in understanding the depth of the discourses she was attempting to navigate   No quantitative data provided |
| Lock, Epston & Maisel (2004) (19) | Asserts that the tenacity of AN is largely due to how it is discursively constructed in medical and psychological settings, meaning that individuals are silenced and unable to story their experience  Exploration of how narrative therapy provides a framework for challenging dominant discourses of AN/BN | N: 2 (Case studies)   - ‘Kris’ (female, 17, diagnosed with AN) - ‘Chloe (female, 19, diagnosed with AN)   Country: New Zealand  Language: English | Narrative therapy  Seen in private practice setting  No further details provided | Qualitative data (as reported by the client/s):   - Lines of enquiry where the self and the problem are intertwined only drive the person further towards identifying with the ED identity/label - Clients reported feeling as though they had to ‘compete’ to be a ‘better anorexic’ than others, when dominant medical discourses were used - The non-stigmatising and non-pathologizing approach offered by narrative therapy was helpful in separating the person from the problem   No quantitative data provided |
| Lock, Epston, Maisel, de Faria (2005) (20) | Exploration of how Foucault’s analysis of power is conceptually important in the development of narrative therapy and how these ideas contribute to anti-AN/BN practices | N: 2 (Case Studies)   - Elizabeth (female, 17, diagnosis of AN) - Kirsten (female, 17, diagnosis of AN)   Country: Unknown  Language: English | Narrative therapy  Individual sessions (number unknown)  No further details provided | Qualitative data (as reported by the client and reflections of therapist):   - Client able to separate themselves from the totalising ED identity - The use of externalising and personifying language is significant in facilitating discussion of the problem’s agendas and tactics   No quantitative data was reported |
| Lundby (2014) (21) | Description of how externalising conversations and double-story development can assist families to talk about problems | N: 2 (Case Studies)   - ‘Even’ (male, 11, seen for ongoing conflict with his brother) - Katie (female, 17, diagnosis of AN)   Country: Norway  Language: Norwegian | Narrative therapy  Sessions involved client and their parent/s (number of sessions unknown)  Seen in private practice setting | Qualitative data (as reported by the client/s):   - Increased ability to identify, define and name the problem, explore its effects, and develop knowledge on how to reclaim their lives from it - ‘Unique outcomes’ were highly significant - Conversations that focused on deconstructing dominant discourse were helpful in positioning them collectively against the problem   No quantitative data provided |
| Maisel, Epston & Borden (2004) (33) | BOOK:  This book stands as a compilation of insider knowledge, poetry and creative writing, excerpts from therapy sessions and analysis from the authors from over 10 years of seeing clients with AN/BN  Explores how the narrative therapy perspective may provide new ways of thinking and speaking about AN/BN and the implications this has for therapeutic practice | Several accounts from female clients are shared throughout the book that highlight their own personal experience with AN/BN, including: Elizabeth (17), Maggie (24), Amy (40), Emily (13), Jennifer (17), Elizabeth (17), Maggie (24), Kirsten (age unknown), Kris (age unknown), Katie (aged mid-thirties), Joeline (38), Riannon (15), Tracy (15), Margaret (‘young adult’), Monique (12), Olivia (34), Laurie (age unknown), Cecily (17), Chloe (age unknown), Emma (13), Victoria (13), Rhoda (39), Lorraine (age unknown), Meg (16), Julie (age unknown), Merrin (‘young woman’), Jessie (age unknown)  Country: Unspecified  Language: English | Narrative therapy  Clients seen for both individual and family therapy across a multitude of settings | Qualitative results (as reported by clients) indicated:   - Recovery was viewed as a shift in a relationship with the ED so that it no longer dominated the client’s life, rather than an absence of symptoms – i.e., reclaiming of life from the ED - Clients were able to take political stances and bequeathed anti-AN writings to the League - Clients obtained meaningful employment, cultivated healthy relationships, graduated from college, remain out of hospital, and continue to use creative writing modalities to engage critically with their ED - Clients reported improved mood, increased self-compassion, a sense of strength and connection with social supports, and development of a more integrated and thickened identity - Clients emphasised the importance of health professionals ‘treating them as a person’, believing in them, and reconnecting them with what is important   No quantitative data provided |
| Nylund (2002) (22) | Example and description of the use of narrative therapy for a client with AN, including the use of post-session letters and poetic writing within narrative framework | N: 1 (Case Study)   - ‘Nannette’ (female, 33, diagnosis of AN)   Country: USA  Language: English | Narrative therapy  Nine individual sessions  Seen in counselling centre setting | Qualitative data (as reported by the client and reflections of therapist):   - Client was able to ‘remember who she was’, reclaim her poetic talents, and access her anti-anorexic voice - Increased connection with family, improved work performance, increased self-compassion and continued engagement in writing poetry - Therapeutic process encouraged and facilitated creativity in the therapist themselves   No quantitative data provided |
| Pedersen (2016) (23) | Explores the use of narrative practices in the treatment of BN in the context of multiple challenges to recovery | N: 1 (Case Study)   - ‘Kiki’ (female, 17, diagnosis of BN, illness duration of two years) - Initial contact with Kiki’s boyfriend George (17); she began attending the following session   Country: Greece  Language: English | Narrative therapy  Combination of individual and couple sessions (total number unknown)  Seen in private practice setting | Qualitative data (as reported by client):   - Client was able to engage in intervention, despite initial hesitancy - Reduced fears surrounding gaining weight - Client found pre-prepared lists useful to remind herself of her accomplishments and skills when she struggled with the problem - Client felt empowered to share her story as a legacy to support others   No quantitative data provided |
| Robbins & Pehrsson (2009) (24) | Proposes alternative treatment model for women with AN, in the context of high dropout rates of current treatment regimes | N: 1 (Case Study)   - ‘Amanda’ (female, 21, diagnosis of AN)   Country: USA  Language: English | Combined narrative and poetry therapy approaches  12 individual sessions  Seen in outpatient treatment centre | Qualitative data (as reported by the client):   - Began discussing feelings of having a personal voice, increased motivation to change, stronger self-efficacy, and decreased desire to engage in self-starvation - Final poem written by client connoted feelings of increased empowerment, hope, and liveliness   No quantitative data provided |
| Russell (2007) (25) | Discusses narrative practices that have been used by the author in working with clients with EDs | N: 1 (Case Study)   - ‘Katerina’ (female, 35, diagnosis of AN and Chronic Fatigue Syndrome)   Country: Australia  Language: English | Narrative therapy  Individual sessions  Private practice setting | Qualitative data (as reported by client):   - Increased ability to name contributing influences that had dictated her sense of identity - Position herself against these discourses - Give expression to her ideas and hopes - Develop alternative stories about her life and identity   No quantitative data provided |
| Scott, Hanstock & Patterson-Kane (2013) (26) | Provides evidence of potential effectiveness of narrative therapy for EDs, presented as an alternative treatment to enhanced cognitive behaviour therapy (CBT-E) | N: 1 (Case Study)   - ‘Victoria’ (female, 28, Caucasian, seen for eating concerns and history of severe AN)   Country: Australia  Language: English | Narrative therapy  10 individual sessions over 12 weeks  Self-referred to rural university psychology clinic | Quantitative data:   - Eating Disorder Risk Scale (EDI-3) completed pre- and post- treatment: results indicated significant decrease on asceticism subscale   Qualitative data (as reported by the client):   - Increased self-care and ability to ‘stand up’ for her needs - Increased assertiveness in other areas of life outside of eating practices - Visualisation of future without ED symptoms - No ongoing significant weight loss or meal skipping   Began to occasionally consume ‘indulgent’ foods |
| Tsun on-Kee (2011) (27) | To explore how narrative therapy can assist when working with individuals struggling with overeating | N: 1 (Case Study)   - ‘John’ (male, age unknown, seen for overeating concerns)   Country: Hong Kong  Language: English | Narrative therapy  Individual sessions (number unknown, at least nine sessions)  Seen in private practice setting | Qualitative data (as reported by the client):   - Increased enjoyment of choice and freedom, separation from self-blame, opening up space for self-acceptance, renewed freedom to choose - Increased sense of agency – reported feeling more determined and capable of altering diet - Identity outside of the problem-saturated story became visible – e.g., descriptions of self as ambitious, hard-working, studious, etc.   No quantitative data provided |
| Weber (2007) (28) | Exploration of the usefulness of an ED assessment and referral service, and how pathologizing or blaming practices can be avoided | Limited details regarding clients – article appears to focus primarily on female clients  Country: Australia  Language: English | Narrative approach  Seen in the context of an ED assessment and referral service  Individual session for purpose of initial assessment and subsequent referrals, with a report provided to the client after the assessment | Qualitative data (as reported by clients):   - The use of the term ‘eating problem’ was appreciated by some, and another client showed a preference for the use of medical terms - Clients expressed the use of externalising language was helpful in creating distance from the problem, and that ‘double-listening’ helped them feel understood - Clients valued having space to tell their story and felt validated and empowered by the report   No quantitative data provided |
| Weber, Davis & McPhie (2006) (29) | To explore the effectiveness of narrative therapy within a group format for dual presentations of depression and eating concerns | N: 7 (Group intervention)   - All females - Aged 20 to 39 years - Duration of lived ED experience ranged from 5 to 23 years - Self-identified as having eating concerns and depression   Country: Australia  Language: English | Narrative therapy  Weekly group therapy program (10 weeks in length, each session was 2.5 hours)  Clients self-referred  Seen in outpatient public health centre | Quantitative data of pre- and post-group tests:   - Reduction in depression scores and eating disorder risk, as measured by the DASS-42 and EDI-3   Qualitative results (as reported by clients and post-group evaluation and supported by survey responses):   - Decrease in harmful daily practices designed to reduce weight, as well as less self-criticism - Use of externalisation and separation from the ED identity was extremely useful |
| White (1986) (30) | Exploration and analysis of family treatment for AN from a cybernetic framework (i.e., that events take a particular course because they are restrained from taking other alternate courses) | N: 1 (Case Study)   - ‘Susan’ (female, 16, diagnosis of AN, 2-year duration of illness)   Country: Unknown  Language: English | Narrative therapy including cybernetics theory  Family therapy context (number of sessions unknown)  Private practice setting | Outcome data is limited; there is some qualitative data that indicated the use of cybernetic questions assisted in provoking new responses and establishing a readiness to change. |
| White (1991) (31) | Exploration of the use of deconstruction in therapy, with reference to case studies | N: 4 (Case Studies)   - ‘Elizabeth’ (female, age unknown, seen for difficulties in relationships with her children) - ‘Amy (female, 23, diagnosis of AN) - ‘John’ and ‘Anne’ (ages unknown, seen for couple therapy) - ‘Robert’ (male, age unknown, seen for abusive behaviour toward his family)   Country: Australia  Language: English | Narrative therapy  Individual and couple sessions (numbers unknown)  Private practice setting | Qualitative data (as reported by clients):   - Elizabeth became better able to connect with her daughters and established healthy mother-daughter relationships - Amy was able to articulate and perform her preferred identity, and took steps to engage others in reclaiming her life - John and Anne resolved their disputes - Robert challenged his abusive behaviour and took responsibility for his actions - Intervention assisted in establishing a sense of agency for clients   No quantitative data provided |
| Zimmerman & Dickerson (1994) (32) | Provides examples of narrative interventions and clinical questions with vignettes | N: 3 (Case Studies)   - ‘Jackie’ (female, 15, diagnosis of AN, three-year illness duration) - ‘Valerie’ (female, 15, diagnosis of AN, five-month illness duration) - ‘Beth’ (female, 21, diagnosis of BN, two-year illness duration)   Country: Canada  Language: English | Narrative therapy  Clients were seen individually and with family members   - Jackie: fortnightly individual sessions for six months - Valerie: one family session, two individual sessions - Beth: 12 weekly individual sessions   Private practice setting | Qualitative data (as reported by clients):   - Jackie – increased assertiveness, improved connection with friends, eating great quantities in different locations, consideration of other identities, obtained employment - Valeria – more confident and assertive, increased food consumption - Beth – felt more in control and powerful over her life, went on to pursue further study   No quantitative data provided |

**REFERENCES**

1. Beaudoin M. Affective double listening: 16 dimensions to facilitate the exploration of affect, emotions, and embodiment in narrative therapy. Journal of Systemic Therapies. 2020;39(1):1-18.

2. Borden A. Every conversation is an opportunity. The International Journal of Narrative Therapy and Community Work. 2007;4:38-53.

3. Brown C. The dangers of trauma talk: Counterstorying co-occurring strategies for coping with trauma. Journal of Systemic Therapies. 2018;37(3):42-60.

4. Brown C, Weber S, Ali S. Women’s body talk: A feminist narrative approach. Journal of Systemic Therapies. 2008;27(2):92-104.

5. Courtney J, Williams L. Linking Lives: Working with women experiencing eating problems. Gecko. 2000:17-38.

6. Craggs T, Reed A. A service-user and therapist reflect on context, difference, and dialogue in a therapy for anorexia. The International Journal of Narrative Therapy and Community Work. 2007;3:30-8.

7. Dallos R. Attachment narrative therapy: integrating ideas from narrative and attachment theory in systemic family therapy with eating disorders. Journal of Family Therapy. 2004;26:40-65.

8. Davidson H, Birmingham CL. Letter writing as a therapeutic tool. Eating and Weight Disorders. 2000;6(1).

9. Dennstedt C. The interplay of substance misuse and disordered eating practices in the lives of young women. The International Journal of Narrative Therapy and Community Work. 2010;3:52-63.

10. Epston D, Morris F, Maisel R. A narrative approach to so-called anorexia/bulimia. In: Weingarten K, editor. Cultural resistances: Challenging beliefs about men, women and therapy. New York: Harrington Park Press; 1995. p. 69-101.

11. Golan M. The journey from opposition to recovery from eating disorders: multidisciplinary model integrating narrative counseling and motivational interviewing in traditional approaches. Journal of eating Disorders. 2013;1(19).

12. Howells K. Narrative work and the metaphor of ‘home’. International Journal of Narrative Therapy and Community Work. 2009;4:32-42.

13. Ibrahim J, Tchanturia K. Patients’ Experience of a Narrative Group Therapy Approach Informed by the “Tree of Life” Model for Individuals with Anorexia Nervosa. International Journal of Group Psychotherapy. 2018;68(1):80-91.

14. Ingamells KM. Wilbur the worrier becomes Wilbur the warrior: A teaching story for narrative family therapists. Journal of Systemic Therapies. 2016;35(4):43-57.

15. Kantor A. Narrative therapy with adolescent females with eating disturbed behavior [Masters]. Canada: The University of Manitoba; 2000.

16. Kraner M, Ingram K. Bursting Out - Breaking Free. A group program for young womsn wanting to reclaim their lives from anorexia nervosa. Gecko. 1997;3:31-57.

17. Lainson KJ. From ‘disorder’ to political action: Conversations that invite collective considerations to individual experiences of women who express concerns about eating and their bodies. International Journal of Narrative Therapy and Community Work. 2016;2:1-15.

18. Lainson KJ. Narrative therapy, neuroscience and anorexia. A reflection on practices, problems and possibilities. International Journal of Narrative Therapy and Community Work. 2019;3:80-95.

19. Lock A, Epston D, Maisel R. Countering that which is called anorexia. Narrative Inquiry. 2004;14(2):275-301.

20. Lock A, Epston D, Maisel R, de Faria N. Resisting anorexia/bulimia: Foucauldian perspectives in narrative therapy. British Journal of Guidance & Counselling. 2005;33(3):315-32.

21. Lundby G. Creating different versions of life: Talking about problems with children and their parents. International Journal of Narrative Therapy and Community Work. 2014;1:18-26.

22. Nylund D. Poetic means to anti-anorexic ends. Journal of Systemic Therapies. 2002;21(4):18-34.

23. Pederson K. Uncovering bulimia’s demanding voice: Challenges from a narrative therapist’s perspective. International Journal of Narrative Therapy and Community Work. 2016;4:1-12.

24. Robbins JM, Pehrsson D. Anorexia Nervosa: A Synthesis of Poetic and Narrative Therapies in the Outpatient Treatment of Young Adult Women. Journal of Creativity in Mental Health. 2009;4(1):42-56.

25. Russell S. Deconstructing perfectionism: Narrative conversations with those suffering from eating issues. International Journal of Narrative Therapy and Community Work. 2007;3:21-9.

26. Scott N, Hanstock TL, Patterson-Kane L. Using narrative therapy to treat eating disorder not otherwise specified. Clinical Case Studies. 2013;12(4):307-21.

27. Tsun on-Kee A. Overeating as a serious problem and foods as real good friends: Revising the relationship with food and self in narrative conversations. International Journal of Narrative Therapy and Community Work. 2011;2:3-15.

28. Weber M. Narrative therapy, ‘eating disorders’, and assessment. International Journal of Narrative Therapy and Community Work. 2007;2:63-70.

29. Weber M, Davis K, McPhie L. Narrative Therapy, Eating Disorders and Groups: Enhancing Outcomes in Rural NSW. Australian Social Work. 2006;59(4):391-405.

30. White M. Anorexia nervosa: A cybernetic perspective. Selected papers. Adelaide: Dulwich Centre; 1986. p. 65-75.

31. White M. Deconstruction and therapy. Dulwich Centre Newsletter. 1991;3:21-40.

32. Zimmerman JL, Dickerson VC. Tales of the body thief. Externalising and deconstructing eating problems. In: Hoyt M, editor. Constructive therapies. London: Guilford Press; 1994. p. 295-318.

33. Maisel R, Epston D, Borden A. Biting the hand that starves you. Inspiring resistance to anorexia/bulimia. New York: Norton; 2004.
